# Pitch–Luminance Crossmodal Correspondence in the Baby Chick: An Investigation on Predisposed and Learned Processes

**DOI:** 10.3390/vision6020024

**Published:** 2022-04-28

**Authors:** Maria Loconsole, Andrea Gasparini, Lucia Regolin

**Affiliations:** 1 Department of General Psychology, University of Padua, 35131 Padua, Italy; andrea.gasparini.5@studenti.unipd.it (A.G.); lucia.regolin@unipd.it (L.R.); 2 School of Biological and Behavioural Sciences, Department of Biological and Experimental Psychology, Queen Mary University of London, London E1 4NS, UK

**Keywords:** crossmodal correspondences, domestic chicks, luminance, auditory pitch, comparative cognition

## Abstract

Our senses are constantly reached by a multitude of stimuli from all different sensory modalities. To create a coherent representation of the environment, we must integrate the various unimodal inputs that refer to the same object into a single multimodal representation. In some cases, however, we tend to bind certain properties of the stimuli without any apparent reason, which is a phenomenon named crossmodal correspondence. For instance, we match a spiky or a rounded shape with the sound “Kiki” or “Bouba”, respectively. Similarly, we associate the left hemispace with low luminance and the right one with high luminance. Instances of crossmodal correspondences were described also in other mammals, and recently, a case of space-luminance crossmodal correspondence was reported in birds (i.e., domestic chicks). Here, we investigate the presence of pitch–luminance crossmodal correspondence in three-day-old chicks, employing experimental methods that exploit either predisposed or learned processes. While failing to report evidence for this phenomenon, we discuss the difference between statistical and structural crossmodal correspondences and the possible role of environmental factors in determining their emergence. Moreover, we discuss the importance of the different experimental methodologies to investigate distinct aspects of this perceptual phenomenon to reach a deeper understanding and unveil the role of innate vs. learned mechanisms.

## 1. Introduction

Our sensory system is constantly bombarded by signals from all different modalities. Thus, to create an integrated representation of the environment, we need to combine information referring to the same stimulus (irrespective of the sensory modality in which it is conveyed) and keep apart the information that refers to different objects or events [1,2]. Multisensory integration bears a strong adaptive value, allowing animals a fast but still accurate analysis of the environmental situation in order to select the most appropriate behavior [2]. Interestingly, there are some cases in which we tend to associate information from different sensory modalities as redundant (i.e., bearing the same meaning) even if there is no explicit reason to do so, namely crossmodal correspondences [1,3]. For instance, we tend to associate lemon scent with a spiky shape and vanilla scent with a rounded shape [4], or high luminance with the right hemispace and low luminance with the left one [5]. This mechanism appears to be universal, as it was attested in populations from different cultures [6,7], and it is probably available at early stages of development, as suggested by studies on preverbal infants [8,9]. A recent line of research focused on the existence of crossmodal correspondences in non-human animals, providing positive evidence in several cases, all within the clade of mammals. For instance, both humans and chimpanzees associate high luminosity with high pitch sounds and low luminosity with low pitch sounds [10]; rhesus monkeys associate spatial proximity with high pitch sounds and further distance with low pitch sounds [11]; dogs associate elevated and low spatial positions, and small and large dimensions, with high and low pitch, respectively [12,13]. This congruous body of evidence suggests that crossmodal correspondences might be a mechanism shared among mammals. Less is known about whether species from other clades might also display a spontaneous mechanism for associating multisensory information. To our knowledge, there is only one study on an avian model (i.e., the domestic chicken) showing visual–spatial crossmodal correspondences that resemble previous evidence on humans. Three-day-old domestic chicks were presented with two identical panels: one on their left, and one on their right. In half of the trials, both panels were dyed black (low luminance condition); in the other half, they were dyed white (high luminance condition). Chicks could circumnavigate either panel to obtain a food reward; however, they showed a preference for the left black panel and for the white right panel [14]. Similarly, adult humans are faster in responding to black squares with their left hand and to white squares with their right hand [5]. One possibility to address the origin of this association could be that of luminance and spatial information being processed by the same brain networks (i.e., structural correspondence [1]). This would be in line with a previous case of multimodal association shared by chicks [15] and humans [16,17], i.e., the SNARC (Spatial–Numerical Association of Response Code) effect, for which we associate small numerosities with the left hemispace and large numerosities with the right one. This effect has been ascribed to a right hemispheric dominance for both visuo-spatial and numerical tasks [15,18,19,20]. If crossmodal correspondences are the result of the neuroanatomical organization of the perceptual system, it is possible to assume that they are shared between different species that resorted to similar brain mechanisms. However, there are some cases of crossmodal correspondences in humans that rely on a semantic component, and for this reason, they are thought to be a prerogative of our species. Semantic correspondences refer to those cases in which the same term is used to indicate two different sensory dimensions, leading to the two dimensions becoming associated. For instance, we could use the terms “high” and “low” both in relationship to luminance (i.e., high luminance as for white, and low luminance as for black) and to auditory pitch (i.e., high pitch as for acute sounds, and low pitch as for low sounds). The fact that these correspondences may reflect a language-based association suggests that they are uniquely human. However, there is evidence of pitch–luminance crossmodal correspondences also in other primates. For instance, both adult humans and chimpanzees show a facilitation effect for which they are faster in responding to a high luminance cue (i.e., a white square) when a high pitch sound is played in the background and to a low luminance cue (i.e., a black square) when the background audio is a low pitch sound [10]. Thus, it might be the case that pitch–luminance association has evolved before the separation between humans and chimpanzees, and, in the case of humans, the association was later translated into language, with the same word referring to both sensory dimensions. As such, rather than a learned or linguistic phenomenon, pitch–luminance association might also represent a basic feature of the sensory system, at least in primates [10].

The present study investigates the presence of pitch–luminance association in the domestic chicken, aiming at providing evidence from a clade distantly related from primates, to better understand the origin of pitch–luminance crossmodal correspondence. To do so, we exploited two different experimental paradigms, to assess either innate predispositions or leaning processes.

## 2. Procedures

In total, 120 male domestic chicks (*Gallus domesticus*, broiler Ross 308) took part in the study (40 subjects in each experiment). We employed birds of one sex only (males) to minimize our sample size. Males were selected, as we used a food reward-based paradigm, for which there is experimental evidence of a higher motivation in male chicks compared to females [21,22]. Fertilized eggs were provided by a local hatchery (Incubatoio La Pellegrina, San Pietro in Gu, PD) and incubated and hatched in the laboratory (FIEM, MG 70/100 FAMILY commercial incubators) at controlled temperature (37–38 °C) and humidity (≈60%). Upon hatching, male chicks were selected (by feather sexing) and housed in pairs in standard metal cages (28 cm large × 32 cm high × 40 cm width). Cages were enlightened by 36 W fluorescent lamps placed 15 cm over the top of the cage from 7 am to 7 pm and followed 2/3 h blocks of dark/light alternation from 7 pm to 7 am. Within the housing cage, chicks had free access to water and food. Chicks were also fed some mealworms (*Tenebrio molitor* larvae) as supplementary diet. Mealworms were also used as food reward in the experiment; thus, chicks that did not show interest in the worms were not included in the procedures. This happened for about 3% of the subjects. Chicks remained housed in pairs with ad libitum food and water until day 3 of life, when they entered the experimental procedures. Hatching and housing conditions remained unvaried between the three experiments.

Each chick was tested individually. The subject was transported in the experimental room within an opaque box. The experimental room was close to the rearing room, and it was kept at the same humidity and slightly lower temperature as the rearing room (27–28 °C). Each chick was gently inserted into the experimental arena where it was left for three minutes to get acquainted to the new environment, which the chick was free to explore, and where some palatable food could be found (see Methods sections for details). This most likely created a positive association with being in the experimental arena, reducing the potential stress of being isolated from the rearing companion. Whenever any sign of excessive stress or discomfort (e.g., distress calls, feather pecking, loss of appetite, reduced motility), the chick was immediately returned to the home cage and excluded from the experiment. However, this was never the case in our study.

At the end of the procedures, chicks were donated to local farmers. All the procedures included in the present study were examined and approved by the ethical committee of the University of Padua (Organismo Preposto al Benessere Animale—O.P.B.A.) and by the National Ministry of Health (Authorization Number: 196/2017-PR granted on 24 February 2017).

## 3. Experiment 1

Experiment 1 included 40 subjects. The whole experimental procedure took place on chicks’ third day of life. Here, we aimed at assessing a spontaneous preference in line with the sound–luminance crossmodal association reported in mammals [10], for which we expected chicks to prefer high luminance (i.e., a white panel) when paired with a high pitch sound (i.e., 1047 Hz), and low luminance (i.e., a black panel) when paired with a low pitch sound (i.e., 175 Hz). For the aim of this study, we decided to employ the same stimuli of the original work by Ludwig et al. (2011) in order to allow a more direct comparison (however, this choice could lead to limitations related to the sensitivity of the different animal models; see General Discussion). The sounds were played at an intensity of 65 ± 5 dB. The luminance and sound characteristics remained unvaried in the three experiments.

Please note that from now on, we will use term “congruent” to refer to the high pitch–high luminance and low pitch–low luminance associations, and we will use the term “incongruent” to refer to the high pitch–low luminance and low pitch–high luminance associations, which is in line with the results reported in humans and chimpanzees [10].

### 3.1. Training

**Figure 1:**
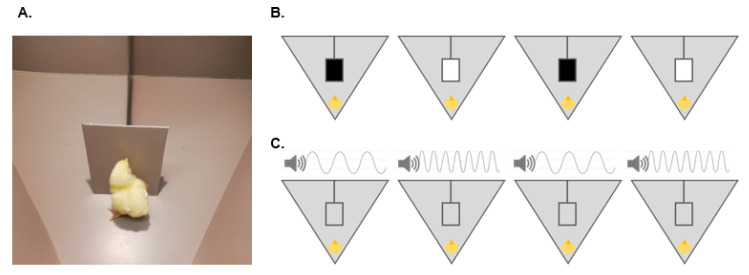
The three phases of the training procedure: (**A**). During the training procedure, a single gray panel is placed in front of the chick. The chick learns to circumnavigate the panel to obtain a small food reward. (**B**). Chicks are familiarized with the black and white panels in four consecutive rewarded trials, in which the two panels are alternated. Half of the chicks start with the black panel (as shown in the example); the other half start with the white panel. (**C**). Chicks are familiarized with high and low pitch sounds in four consecutive rewarded trials. In this case, the gray panel is used to avoid associating luminance and sound prior to test. Half of the chicks start with the low pitch (as shown in the example), while the other half start with the high-pitch sound.

Training lasted between 15 and 20 min per chick and it was identical for Exp. 1 and Exp. 2. The aim of the training was acquainting the subject to circumnavigating a vertical panel to retrieve a small food reward (i.e., half a mealworm hidden behind the panel). The experimental arena consisted of a plastic triangle (76 cm side × 32 cm high wall) lined with 290 gr opaque gray contact paper (opaque polyvynil cloryde). The arena was enlightened by a 40 W lamp placed approximately 80 cm above the center of the experimental apparatus. In one corner of the arena, a removable glass partition created the starting position in which the chick was confined before the beginning of each trial. A vertical partition (25 × 37 cm) divided the side opposite to the starting position in two separate and mirrored (left and right) areas. During training, a single gray panel was placed in front of the starting position, at the end of the vertical partition, and orthogonal to it (Figure 1A). During this phase, the vertical partition was irrelevant for the task, but it would be used later to unequivocally score the direction chosen by the chicks on each trial of the test (see Section 3.2). In the initial phase of training, the chick was placed in the arena and let immediately free to explore it for two to three minutes. After the chick found and consumed the food reward hidden behind the gray panel, it was gently removed from the arena and placed in a holding box, while the experimenter placed a new food reward behind the panel. Then, the chick was inserted in the starting position, and a new trial was started. Once the chick seemed comfortable with this procedure, a short period of confinement within the starting position (through a transparent partition) was introduced before each trial. After a few seconds, the partition was lifted, and the chick was let free to walk around. The procedure was repeated until the chick promptly circumnavigated the panel in three consecutive training trials. Then, the chick underwent four additional trials, in which the central gray panel was substituted by either a white (170 hue, 0 saturation, 255 lightness) or a black (170 hue, 0 saturation, 0 lightness) panel in alternating order (Figure 1B). This was needed to familiarize the chick to the panels to be used for testing and to allow it to associate each such panel with food. During this phase, no multimodal information was provided (i.e., there was always a single panel in the center of the arena but no auditory information). The familiarization with the white and black panels also allowed to reduce the possibility of chicks, creating a specific luminance–reward association on the very first test trials, which could have biased the following choices. Lastly, the chicks were familiarized also with the auditory stimuli. To this aim, we used the gray panel, which was baited with half a mealworm, for four training trials during each of which a sound (either high or low pitch, in alternating order) was played (Figure 1C). The auditory track was played by a small speaker (SONY SRSXB01, 8 × 5 × 5 cm) placed centrally on top of the arena, above the vertical partition.

### 3.2. Test

**Figure 2:**
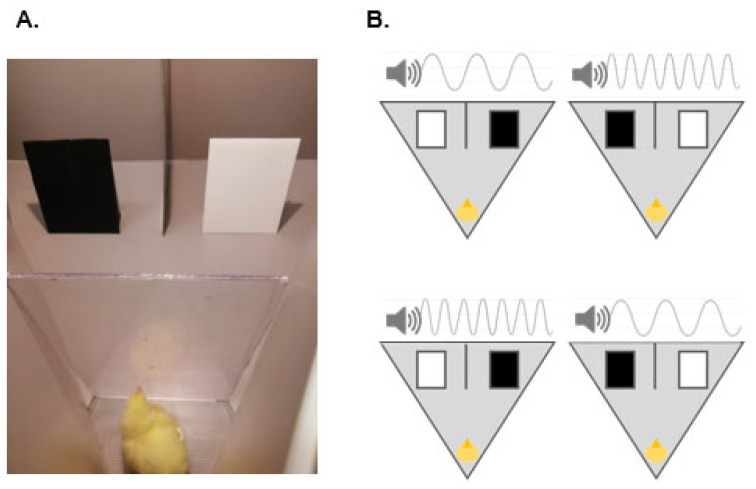
Test: (**A**). The chick restrained in the starting position can see both panels: one on the left (in this example, the black one), and one on the right (in this example, the white one) of the vertical partition. When the glass partition is removed, the chick is let free to approach either panel. The trial is ended when the chick circumnavigates either panel with at least 2/3 of its body, including the head. (**B**). Each chick undergoes 20 consecutive trials, in which 4 possible combinations are alternated in semi-random order (i.e., the same combination never occurs more than twice in a row. The trials differ for the spatial position of the black and white panel (i.e., left, or right) and for the pitch of the background audio (i.e., high, or low).

Test took place immediately after training and in the same arena. In this case, however, two panels were visible at once, on the two sides of the vertical partition, equidistant from the vertical partition (approximately 7 cm), from the walls of the arena (approximately 10 cm), and from the speaker above it (approximately 40 cm). One of the panels was black, and the other was white (Figure 2). The spatial position of the panels (left/right) was counterbalanced between trials. The entire experimental procedure was video-recorded by a camera placed 30 cm on top of the arena. At the beginning of each trial, the chick was gently placed by the experimenter in the starting position, behind the glass partition. The experimenter then moved away from the arena to a defined spot out of the chick’s sight and remained still for 3 s; then, it lifted the glass partition. In this way, the experimenter was blind with respect to the chick’s body orientation when starting the trial. To control for other possible biases, the experimenter remained out of the chick’s sight for the entire duration of the trial, observing its behavior from the camera. The experimenter interacted again with the chick only once the trial was completed: to move it to the transport box while preparing the setup for the subsequent trial. Each subject was tested in 20 consecutive trials in which it was let free to approach either of the two panels. Importantly, both panels were always equally baited. After the chick had circumnavigated one panel and ate the associated reward, it was gently removed from the arena, without the possibility of approaching the other panel. The vertical partition maintained the chick within one choice area (i.e., close to the chosen panel), while the experimenter gently removed the subject from the arena. Then, a new trial was started. In each trial, a background audio was played. This was the high-pitch sound for half of the trials and the low-pitch sound for the other half. The alternation between high-pitch and low-pitch sounds among trials followed a semi-random order, for which the same sound never occurred in more than two consecutive trials. Similarly, we pseudo-randomized the position of the black and white panels between trials, so that the same panel never occurred in the same spatial position for more than two consecutive trials. For each trial, the panel (i.e., black or white) chosen by the chick was scored by the experimenter. We considered as a choice the circumnavigation of the panel with 2/3 of the body, including the head of the chick.

### 3.3. Data Analysis and Results

Data were analyzed using the statistical software R. We employed a generalized linear mixed-effect model (R package:lme4 [23]) with a binomial structure, with the dependent variable being dichotomous (i.e., choice of the black or of the white panel). The independent variable was the background auditory stimulus (i.e., high pitch or low pitch). Subject ID was included as a random effect. We ran a post hoc analysis with Bonferroni correction in order to determine the direction of the effect of the predictors (R package: emmeans [24]).

**Figure 3:**
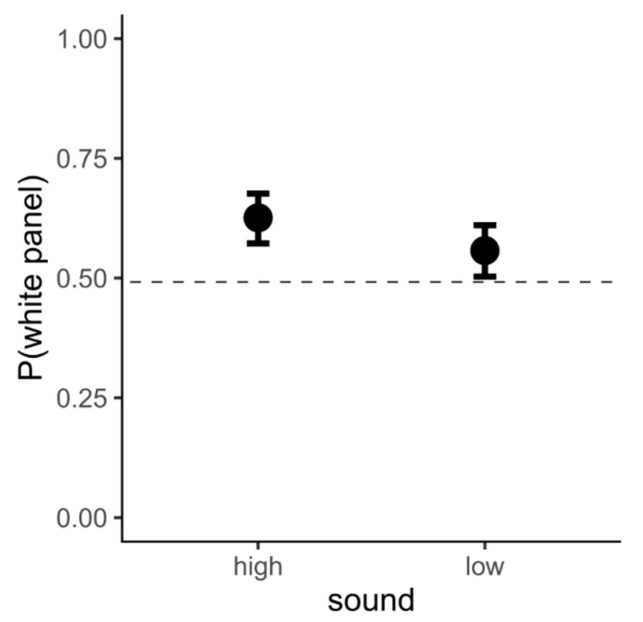
Results from Exp. 1: In both cases (high-pitch and low-pitch background audio), chicks preferred the white panel. On the *y*-axis is the probability of choosing the white panel. The bars indicate 95% confidence intervals.

Contrary to our initial hypothesis, we did not find an effect of the sound on the preference for luminance (X^2^ = 3.804, *p* = 0.051). Post hoc analysis revealed a preference for the white panel in both the high pitch (P(white panel) = 0.626, SE = 0.027, z = 4.524, *p* < 0.0001) and the low pitch conditions (P(white panel) = 0.557, SE = 0.028, z = 2.07, *p* = 0.039) (Figure 3). There was no statistically significant difference between the two conditions (contrast(high/low pitch) = 1.33, SE = 0.193, z = 1.95, *p* = 0.051).

### 3.4. Interim Discussion, Experiment 1

In the original study on humans and chimpanzees by Ludwig et al. (2011), subjects were required to categorize stimuli presented on a monitor that could be of high or low luminance (i.e., white or black squares). During the task, a background sound was played, it being of either a high or low pitch. Participants’ performance was influenced by the background audio, and they performed better (i.e., they were faster and more accurate) in responding to white squares when listening to the high-pitch sound and to black squares when listening to the low-pitch sound [10]. This facilitation was interpreted as a pitch–luminance crossmodal correspondence, which is shared between humans and chimpanzees. The fact that also chimpanzees possess such a correspondence rules out the possibility of this phenomenon having a semantic origin (as previously postulated [1]). However, it is yet to be determined whether it is limited to primates or if it is also present in more distantly related species. To this aim, in Exp. 1, we tested baby chicks, employing the same paradigm of playing a background sound while the chicks were performing a free choice task. As crossmodal correspondences are thought to be a spontaneous phenomenon that occurs in the absence of training, we decided to employ a free-choice task rather than training the chicks to choose either stimulus. After a short preliminary phase needed to acquaint the chicks to circumnavigate a neutral (gray) panel to obtain a food reward, we presented chicks with two panels: one dyed black and one dyed white. We hypothesized that the chicks’ choice of either panel would be influenced by the background audio, specifically that they would have preferred the white panel when hearing a high-pitch sound and the black panel when hearing a low-pitch sound. However, while qualitative observations may suggest a tendency in the expected direction, there was no statistically significant effect. Unfortunately, a null result does not allow any conclusion, as other factors might have occurred in dampening the crossmodal effect. In particular, we hypothesized that some aspects of our paradigm might have been extremely cognitively demanding for the baby chicks. When chicks were tested with the space–luminance crossmodal correspondence, they underwent a similar paradigm; however, in each trial, they were shown two identical panels, and between trials, they experienced changes in only one dimension (i.e., the luminance of the two panels). In the case of the present study, chicks were required to process two different stimuli at the same time (i.e., in each trial, they saw both the white and the black panel) with trial-to-trial changes in both the spatial position of the panels and the background audio. A cognitive overload might have caused a failure in processing the information coming from one sensory modality, thus making the emergence of the crossmodal correspondence effect impossible. To test for this possibility, we designed a second experiment in which we kept one sensory dimension constant across trials. We divided the test in two blocks of 10 trials each; in each block, we always presented the same auditory stimulus and only changed the left/right position of the two panels. This procedure should significantly reduce the cognitive effort required to the chicks for solving the task.

## 4. Experiment 2

### 4.1. Training

Experiment 2 included 40 subjects. The whole experimental procedure took place on chicks’ third day of life. The training procedure remained unvaried with respect to Experiment 1 (see Section 3.1). As for Experiment 1, once a chick completed the training, it immediately entered the test phase.

### 4.2. Test

The testing procedure was the same as described for Experiment 1 (see Section 3.2), the only difference being that now the new sample of chicks underwent two consecutive blocks of 10 trials each, one with the high-pitch background audio, and the other with the low-pitch background audio. The pitch (high or low) presented in the first block was counterbalanced between subjects.

### 4.3. Data Analysis and Results

As for Experiment 1, we employed a generalized linear mixed-effect model (R package:lme4 [23]) with a binomial structure, with the dependent variable being dichotomous (i.e., choice of the black or white panel). We included in the model as an independent variable the background auditory stimulus (i.e., high pitch or low pitch) and the trials (to check for the effect of being exposed to the same sound over multiple consecutive trials). Subject ID was included as random effect. We ran a post hoc analysis with Bonferroni correction in order to determine the direction of the effect of the predictors (R package: emmeans [24]).

**Figure 4:**
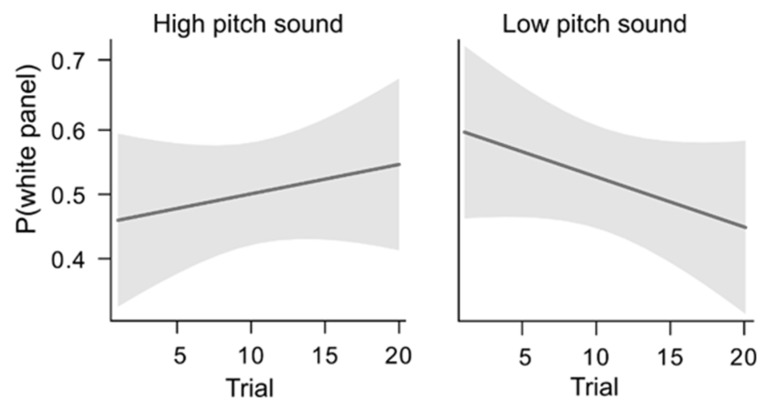
Results from Experiment 2: Chicks underwent 10 consecutive trials with high pitch and 10 consecutive trials with low-pitch background audio. In both cases, there was no significant preference for a specific luminance (i.e., white vs. black panel). On the *y*-axis is the probability of choosing the white panel. The shaded area indicates 95% confidence intervals.

Similar to Experiment 1, we did not find any effect of the sound on the preference for luminance (X^2^ = 0.272, *p* = 0.602). In addition, we did not find any effect of trials (X^2^ = 0.245, *p* = 0.621) nor of the interaction between the background sound and the trials (X^2^ = 1.637, *p* = 0.201). There was no significance when considering the high background trials (trend(white panel) = 0.019, SE = 0.024, z = 0.786, *p* = 0.432) nor when considering the low background trials (trend(white panel) = −0.032, SE = 0.024, z = −1.339, *p* = 0.181) (Figure 4). In addition, there was no statistically significant difference between the two conditions (estimated difference(high–low pitch) = 0.05, SE = 0.039, z = 1.279, *p* = 0.201).

### 4.4. Interim Discussion, Experiment 2

Exp. 1 and Exp. 2 both failed at showing pitch–luminance crossmodal correspondences in baby chicks. Yet, in both cases, a descriptive approach would suggest the presence of a faint tendency to associate high luminance with high-pitch sounds and low luminance with low-pitch sounds. In Experiment 2, we tested chicks in blocks of 10 trials each, in which the trials differed for the spatial position of the two panels (left or right) but were always accompanied by the same audio (high or low pitch). This was aimed at excluding the possibility that chicks in Exp. 1 had failed because of the task being too cognitive demanding (i.e., in Exp. 1, both the spatial position of the panels and the background audio changed from trial to trial). Unfortunately, even when reducing the complexity of the task, we failed in showing pitch–luminance crossmodal correspondences in baby chicks. There might be the possibility of other factors affecting chicks’ performance and contrasting the emergence of the crossmodal association. In particular, one downside of this procedure is that by keeping the auditory stimulus constant across a block of 10 trials, chicks might habituate and cease to process it as a dimension, resulting in the loss of the association [25,26]. Another factor that should be taken into consideration is the fact that a spontaneous choice task might not be sensitive enough for detecting the hypothesized effect. Subtle preferences which are not expressed in free choice preferential tasks could still be detected in a supervised learning paradigm, as a facilitation in the acquisition of a positive associations with the preferred stimulus, as compared to the non-preferred stimulus.

To overcome these limits, we designed a third experiment comprising an acquisition and a reversal learning phase. In the acquisition separate groups of chicks were food-reinforced to associate a black (or a white) stimulus with a high (or low) auditory sound. There were four separate conditions, two congruent (i.e., the high and low luminance must be associated with the high and low pitch, respectively) for which we expected a facilitated acquisition and a more difficult reversal, and two non-congruent (i.e., the high and low luminance must be associated with the low and high pitch, respectively) for which we expected the opposite. 

## 5. Experiment 3

### 5.1. Subjects

Experiment 3 included 40 subjects. Acquisition took place on day three of life, whereas reversal was run the following day, when chicks were four days old. The hatching and rearing conditions were the same as described for Experiment 1 and Experiment 2 (see Procedures). However, in this case, the new sample of 40 chicks was randomly divided into four different groups, i.e., 10 chicks per each possible condition: (i) Congruent acquisition with white panel; (ii) Congruent acquisition with black panel; (iii) Incongruent acquisition with white panel; (iv) Incongruent acquisition with black panel. The conditions are described in detail in Section 5.3 and Section 5.4.

### 5.2. Training

The experimental arena was the same as described for Experiment 1 and Experiment 2, the only difference being that in this case, in front of the starting position was a gray plastic box (7.5 cm side × 4 cm height), hiding a small drawer (6.5 cm side × 3.5 cm height) that could be open by the experimenter via a hidden mechanism. Chicks were trained to peck on the box to obtain the reward (i.e., half a mealworm) contained inside the drawer. Each chick was trained individually via a shaping procedure, in which the worm was initially visible and then progressively hidden inside the drawer. The shaping ended when upon being released in the arena, the chick walked straight to the box, pecked onto it to obtain the opening of the drawer, and retrieved the worm in three consecutive trials. Acquisition took place immediately after the end of shaping.

### 5.3. Acquisition

**Figure 5:**
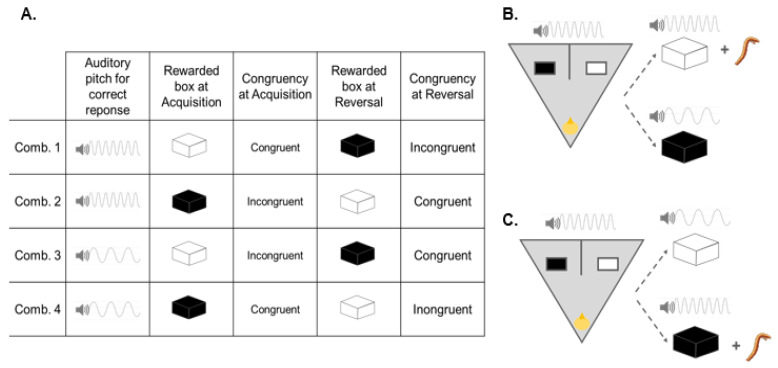
The experimental paradigm used in Experiment 3: (**A**). Chicks were randomly assigned to one of four possible combinations, varying for the luminance of the rewarded box at acquisition (and therefore at reversal) and for the pitch of the sound associated with the reward. In Comb. 1 and Comb. 4, the acquisition presented an association congruent with the pitch–luminance correspondence reported in humans and chimpanzees, while the association became incongruent at reversal. We hypothesized that reversing these two conditions would be harder, as it required chicks to disregard the previously reinforced congruent association and learn an incongruent one. In Comb. 2 and Comb. 3, the acquisition requires an association opposite to the pitch–luminance correspondence reported in human and chimpanzees. However, the association shifts to congruent at reversal. We hypothesized that these two conditions would be easier at reversal, as the chicks are required to disregard an incongruent association in favor of a congruent one. (**B**). Example of the acquisition phase (Comb. 1). (**C**). Example of the reversal phase (Comb. 1).

During acquisition, the chick was presented with two boxes identical except for the color: one black and one white, each placed on either side of the vertical partition. The position (left or right) of each box was pseudo-randomized among trials, so that the same box never occurred in the same spatial position for more than two consecutive trials. Each chick was randomly assigned to one of four possible conditions of rewarded stimulus plus sound associated to the correct response (Figure 5A). Importantly, chicks were required to peck on the correct stimulus, and the auditory track was played at the same time of their pecking. Temporal co-occurrence has been shown to represent a crucial factor in strengthening multimodal association between visual and auditory stimulation in humans [27,28], and it also constitutes a well-known facilitation in animals’ associative learning and conditioning [29,30]. In this way, we aimed to hardwire the luminance–sound association and at the same time obtain direct evidence of chicks paying attention to the stimuli (i.e., by setting a learning criterion). The chick was placed in the starting position in the triangular arena, behind the glass partition. Before the glass partition was lifted, the sound to be associated with the correct choice (e.g., high pitch) was played. In front of the starting corner, there were two boxes: one on the left and one on the right of the vertical partition, one dyed black and one dyed white. The spatial position (left or right) of the boxes varied according to a semi-random sequence (i.e., the same box never appeared in the same position more than twice consecutively). When the chick was released in the arena, it was free to interact with either box. The trial was considered completed when the chick pecked on one of the two boxes. If it pecked on the rewarded box, the trial was scored as “correct”. If it pecked on the unrewarded box, the trial was scored as “incorrect”. If it pecked on the correct box (e.g., the white box), the associated auditory sound was immediately played (in this example, the high pitch sound) while the box was opened, allowing the chick to eat the reward from the drawer. If the chick pecked on the wrong box (in this example, the black box), the other auditory sound was played (in this example, the low pitch sound) and the box was opened; however, in this case, the drawer was empty (Figure 5B). The chick was then gently removed from the arena, and a new trial was started. The acquisition was considered completed upon reaching a passing criterion of 17 correct trials out of 20 consecutive trials. After acquisition, the chick was placed in the home cage with its social companion, food, and water, until the next day, when it entered the reversal phase. Even though this is a relatively easy task for the chicks (i.e., based on associative learning and simple discrimination), we hypothesized that there could be a difference for which chicks trained with a congruent sound–luminance association may require fewer trials to complete acquisition than chicks trained with the incongruent association.

### 5.4. Reversal

The procedure at reversal was identical to the one used for acquisition. Again, the position (left or right) of each box was pseudo-randomized across trials, so that the same box never occurred in the same spatial position more than twice consecutively. At reversal, though, the rewarded box was the previously unrewarded one, and, vice versa, the box that had previously led to a reward was now unrewarded. Thus, if the chick had learned during acquisition to peck the white box, at reversal, it should learn to disregard the previous association and choose the black box (Figure 5C). To ensure that chicks remembered the acquisition, we excluded those subjects that on the very first trial of the reversal did not peck the box rewarded during acquisition. This happened for five subjects. The reversal was considered completed upon reaching the passing criterion of 17 correct out of 20 consecutive trials. As for the acquisition, we hypothesized a facilitation effect for chicks required to learn the congruent rather than incongruent association. Moreover, in the incongruent reversal, chicks would also be required to abandon a previously acquired congruent association in favor of an incongruent one (potentially resulting in an increased number of trials to criterion). Vice versa, in the congruent reversal, chicks previously trained with an incongruent association might find it easier to abandon it for a new (congruent) associative rule.

### 5.5. Data Analysis and Results

Data were analyzed using a generalized linear mixed-effect model (R package:lme4 [23]) with a Gaussian structure. We included in the model as a dependent variable the number of trials needed to reach the passing criterion. Independent variables were the session (i.e., acquisition or reversal), the congruency (see Figure 5A), and their interaction. Subject ID was included as a random effect.

In addition, we ran a generalized linear mixed-effect model with a binomial structure for both acquisition and reversal, in which the chicks’ response on each trial (i.e., correct, or incorrect) was included as a dependent variable, and independent variables were the congruency (i.e., congruent or incongruent) and the spatial position of the correct stimulus (i.e., left, or right). In fact, a previous work showed that chicks exhibit a facilitation effect in the reversal phase when the correct stimulus is positioned in the left hemispace. This is probably related to a right hemispheric specialization for the cognitive mechanisms involved in the task, such as novelty detection, visual attention, and more in general, behavioral flexibility [31].

For both analyses, we ran a post hoc analysis with Bonferroni correction in order to determine the direction of the effect of the predictors (R package: emmeans [24]).

**Figure 6:**
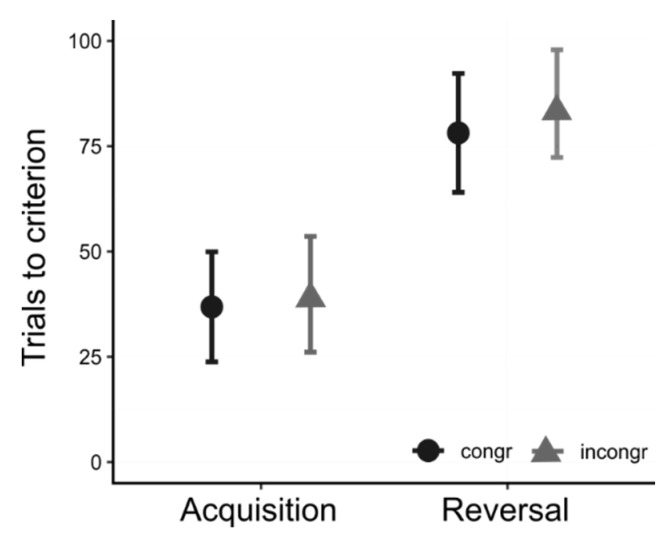
Results of Exp. 3: Chicks required significantly more trials (about 40) to complete the reversal than the acquisition. The congruency of the association did not seem to affect performance. In the incongruent conditions, chicks required on average 3 extra trials in the acquisition and 6 extra trials in the reversal; these differences were not significant.

When considering the total number of trials to criterion (Figure 6), we found a significant effect of the session (X^2^ = 48.886, *p* < 0.0001) but no effect of the congruency (X^2^ = 0.636, *p* = 0.425) nor of the interaction between session and congruency (X^2^ = 0.075, *p* = 0.785). Post hoc analysis showed that chicks required significantly more trials in the reversal compared to acquisition (estimate difference(acquisition-reversal) = −43.28, SE = 6.2, t = −6.981, *p* < 0.0001). For what concerns the hypothesized congruency effect, it is possible to notice that on average, chicks needed more trials to reach criterion in the incongruent condition with respect to the congruent condition for both acquisition (estimate difference(congruent–incongruent) = −3, SE = 9.53, t = −0.315, *p* = 1) and reversal estimate difference(congruent–incongruent) = −6.94, SE = 9.56, t = −0.725, *p* = 1); however, the difference does not appear to be statistically significant.

**Figure 7:**
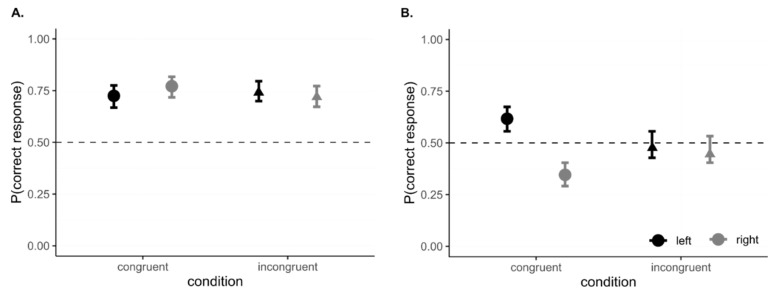
Results of the side bias analysis in Experiment 3: (**A**). Acquisition. Chicks perform equally well in both the congruent and incongruent conditions (on the x axis), irrespective of the spatial position of the correct box. (**B**). Reversal. Chicks’ performance in the congruent condition is affected by a spatial bias, for which subjects tend to respond correctly to the left hemispace but not to the right one. In the incongruent condition, however, chicks do not exhibit such a spatial bias, and chicks performed at chance level irrespective of the spatial position of the correct box. In light gray, chicks’ performance when the correct response must be provided in the right hemispace, in black, chicks’ performance when the correct response must be provided in the left one.

When analyzing the effect of the spatial position of the correct response during acquisition, we did not find any significant effect (all *p* > 0.05). Post hoc analysis shows that chicks’ performance was similar for both congruent and incongruent trials, irrespective of the spatial position of the correct box. At reversal (Figure 7), we did not find an effect of the congruency (X^2^ = 0.0003, *p* = 0.986); however, we found a significant effect of the side (X^2^ = 68.828, *p* < 0.0001) and of the interaction side x congruency (X^2^ = 48628, *p* < 0.0001). Post hoc analysis shows that chicks in the congruent condition exhibited a left-spaced bias (contrast (Left/Right) = 3.049, SE = 0.315, z = 10.798, *p* < 0.001), whereas in the incongruent condition, they equally responded to both sides (contrast (Left/Right) = 1.101, SE = 0.114, z = 0.927, *p* = 1).

### 5.6. Interim Discussion, Experiment 3

Experiment 3 aimed at exploring pitch–luminance crossmodal correspondences in a reversal learning task, hypothesizing that performance could be affected by the visual and auditory stimuli being congruent (or incongruent) with the crossmodal association. By comparing chicks’ performance at acquisition, we could assess whether learning was facilitated when the luminance of the box was matched with the congruent sound (e.g., a correct response for the black box matched with a low-pitch sound). Chicks took on average three trials more when acquiring an incongruent rather than a congruent association. Such difference was not significant. The acquisition phase could have been relatively simple for the chicks, and a ceiling effect might explain such null results. For this reason, we looked at the reversal phase, in which the same chicks were required to learn the opposite association (e.g., if they had learned to respond to the black box, now they must respond to the white box to obtain the reward). Chicks were hence required to disregard the previously learned rule to acquire the new one, and this makes the task more difficult. At reversal, chicks that had to learn the incongruent condition took on average seven more trials to reach the criterion as compared to the chicks in the congruent condition. However, this difference does not reach statistical significance. We also tested for any effect of the spatial position of the correct box, as in a previous study that employed a reversal learning task, chicks had shown a left space bias [31]. Our results were in line with those of the previous study only when chicks experienced a pitch–luminance congruent reversal (i.e., high luminance matched with high pitch or low luminance matched with low pitch). In this case, chicks provided a correct response only when they were required to select the box in the left hemispace, suggesting a main activation of the right hemisphere in this task. In birds, hemispheric dominance can be inferred from lateralized behaviors. This is due to the peculiar anatomical structure of birds (i.e., they possess a virtually complete decussation of the optic chiasm, for which each eye projects almost exclusively to the contralateral hemisphere, and they lack a corpus callosum that connects the two hemispheres) [32,33,34,35]. The leftward bias previously described in reversal learning has been thought to reflect a right hemispheric activation in terms of novelty detection [35,36] and visuo-spatial attention [37]. Both mechanisms are crucial for making sense of the environmental changes that could account for the previously acquired response suddenly not leading any longer to the expected reward. In spite of the lack of direct evidence of the pitch–luminance correspondence in the baby chicks, the difference in the lateralized behavior between congruent and incongruent conditions is suggestive. While the behavior in the congruent conditions seems in line with previous studies that employed the reversal learning paradigm, incongruent conditions appear to trigger a different processing. One possible interpretation is that of the incongruent task not triggering the aforementioned right-hemispheric mechanisms (i.e., visuo-spatial attention and novelty detection). An alternative hypothesis could be that of the incongruent condition somehow triggering also a left hemispheric activation, which counterbalanced the right-hemispheric bias. In fact, a left hemispheric activation was reported in birds for tasks that require inhibitory control [36,38], which in the case of the current study can be strengthened by the necessity of inhibiting a congruent response code to favor an incongruent one. This is also consistent with studies on humans that showed a higher activation of the left hemisphere when subjects were presented with a conflict between information (e.g., in a Stroop task) [39].

## 6. General Discussion

Humans show a shared tendency to associate high luminance and high pitch sounds in the absence of an apparent rule to do so [10,40,41]. One hypothesis is that of the two sensory modalities sharing some semantic features; i.e., it is possible to describe polarized levels of both luminance and pitch with the words “low” and “high” [1]. However, this hypothesis was critically dampened by recent evidence of a similar association in chimpanzees, which suggests that the effect is not a human prerogative [10]. Yet, it is still unknown whether it is shared only within mammals or whether, as for space–luminance correspondence [14], it is widespread to other clades. The present study aimed at answering this question by testing pitch–luminance crossmodal correspondences in the domestic chick (*Gallus domesticus*). Providing evidence from a precocial bird species would allow for a better understanding of this phenomena from a both phylogenetic and ontogenetic point of view. Unfortunately, our results are not conclusive, as we failed in showing the pitch–luminance correspondence in our animal model. In spite of this, our data offer some descriptive observations that are worthy to discuss, as they can provide some insights for further investigations. It is possible that chicks do possess pitch–luminance correspondence, but that this was weakened by some external factors, to the point that the association could not be detected by our behavioral paradigms. A critical point is indeed the ecological validity of the association for the baby chicks. Even though they were tested at a very early age (i.e., 3 days old), they had extensive experience prior to test of both pitch and luminance information. This is because during rearing, they were exposed to salient cues (own and other chicks’ visual, acoustic, and tactile cues), which likely offered selective experience about high luminance (i.e., the light-colored fluff of this strain of baby chicks) and high-pitch sounds (i.e., the calls and the tweets of the baby chicks). We must consider the possibility that this experience biased the chicks’ preferences, weakening the processing of other combinations of pitch and luminance. In addition, it might also be the case of this experience further strengthening chicks’ greater sensitivity to the high-pitch sound. In fact, as this study aimed at replicating the visual–auditory crossmodal association previously found in humans and chimpanzees [10], we decided to employ the same stimuli as in the original study by Ludwig (2011). This could have allowed a more direct comparison between studies; however, it may have had a negative impact on the ecological validity of the stimuli. Future studies can lean on our proposed methodology to test chicks with sounds comprised in the chicks’ most sensitive window of 600–2500 Hz. The paradigm could be further refined by also employing naturalistic stimuli, such as conspecifics’ calls, to which chicks show a much stronger reaction [42]. Employing more naturalistic stimuli should also include the test of female chicks, which are very responsive to affiliative signals and to social information [21,22,43].

Another relevant factor might be the age of the subjects. Even though chicks did show a case of multimodal association already in their first week of life [14,15], there might be the possibility of pitch–luminance association emerging at later stages of life. In fact, in humans, this kind of association appears to be related to language acquisition, hinting at a later development of this phenomenon [1,3]. Further studies could expand on the current work, investigating whether it is possible to observe a developmental trajectory in the emergence of pitch–luminance crossmodal correspondence, testing chicks at different stages of life.

Overall, while our results do not support the presence of pitch–luminance crossmodal correspondences in chicks, they provide some observations that might guide future investigation in reaching a deeper understanding of this phenomenon by providing insights and suggestions from both theoretical and methodological points of view.

## Data Availability

The original dataset is available upon request to the corresponding author.
